# Symptomatology and Clinic of Hydronephrosis Associated With Uretero Pelvic Junction Anomalies

**DOI:** 10.3389/fped.2020.00520

**Published:** 2020-09-30

**Authors:** Ilmay Bilge

**Affiliations:** Division of Pediatric Nephrology, Department of Pediatrics, School of Medicine, Koc University, Istanbul, Turkey

**Keywords:** ureteropelvic junction, hydronephrosis, urinary tract infection, pain, children

## Abstract

The most common cause of hydronephrosis in the pediatric age group is ureteropelvic junction-type hydronephrosis (UPJHN). Since the advent of widespread maternal ultrasound screening, clinical presentation of hydronephrosis associated with UPJ anomalies has changed dramatically. Today most cases are diagnosed in the prenatal period, and neonates present without signs or symptoms. For those who are not detected at birth, UPJHN eventually presents throughout childhood and even adulthood with various symptoms. Clinical picture of UPJHN highly depends on the presence and severity of obstruction, and whether it affects single or both kidneys. Abdominal or flank pain, abdominal mass, hematuria, kidney stones, urinary tract infections (UTI), and gastrointestinal discomfort are the main symptoms of UPJHN in childhood. Other less common findings in such patients are growth retardation, anemia, and hypertension. UTI is a relatively rare condition in UPJHN cases, but it may occur as pyelonephritis. Vesicoureteric reflux should be kept in mind as a concomitant pathology in pediatric UPJHN that develop febrile UTI. Although many UPJHN cases are known to improve over time, close clinical observation is critical in order to avoid irreversible kidney damage. The most appropriate approach is to follow-up the patients considering the presence of symptoms, the severity of hydronephrosis and the decrease in kidney function and, if necessary, to decide on early surgical intervention.

## Introduction

Widespread use of prenatal ultrasonography (US) gave clinicians the opportunity to diagnose urinary tract abnormalities much earlier and more frequently than the past (1). The approximate varying incidence of 1 per 750–2,000, ureteropelvic junction type hydronephrosis (UPJHN) is the most common cause of childhood hydronephrosis (2). It occurs in 13% of children with prenatally detected renal pelvis dilatation, and is more common on the left side, more common in boys (2:1- male to female), and is rarely seen bilaterally (2–4).

An obstruction at ureteropelvic junction level which is defined as restriction of urine outflow from pelvis renalis to the ureter may result in progressive deterioration or hinder normal renal development (5–8). Over 50% of all cases considered to have kidney abnormalities in the prenatal period are hydronephrosis, but unfortunately there are currently no reliable prenatal diagnostic test that can distinguish obstructive hydronephrosis from non-obstructive (8–10). The differentiation between urinary tract obstruction and dilatation is the most important problem in the management of these patients (6, 11, 12). Since the clinical course are quite diverse, and generalization is rather difficult, the most appropriate approach of UPJHN seen in children would be to evaluate on a patient basis (4, 13–16).

In this review, the purpose is to provide general information about the clinical presentation and symptomatology of hydronephrosis associated with uretero pelvic junction anomalies, as well as discussing the clinical findings through some case examples.

## Clinical Presentation

Over the last decades, clinical presentation of patients with UPJHN has shifted from the “symptomatic” patients to the “asymptomatic” neonates who present with prenatal diagnosis (1–4, 15, 16). UPJHN cases without a prenatal diagnosis present with various symptoms such as febrile urinary tract infection (UTI), abdominal masses, pain, pyuria, hematuria, and some gastrointestinal symptoms in the post-natal period or later years. Failure to thrive, anemia, hypertension, and urinary extravasation are much more rare symptoms of UPJHN in childhood (14–16).

Clinical picture of hydronephrosis associated with uretero pelvic junction anomalies highly depends on the presence and severity of obstruction, and whether it affects single or both kidneys. However, most infants with severe hydronephrosis are otherwise asymptomatic and rarely require intervention during follow-up (6, 8, 12). Therefore, parallel to the change in its clinical presentation, the first enthusiasm for early intervention of hydronephrosis associated with UPJ anomalies has turned into a more conservative approach in recent years (11, 15, 17–19). Although there are numerous publications regarding conservative management of UPJ hydronephrosis, and the current trend is to follow the infants through clinical and US findings, the general practice shows a wide variety even today (20–26).

The most accurate answers to the questions of which treatment is better for symptomatic infants, which kidney will benefit from surgery and which patients should be followed up expectantly are still not clear. There are two issues that do not have much discussion during follow-up period of UPJHN patients. First; close monitoring is mandatory for high-grade hydronephrosis managed conservatively; secondly, severe hydronephrosis suggesting an obstruction in solitary kidney is an indisputable condition that requires urgent intervention. An urgent intervention may also be required in patients presenting with urosepsis or acute renal failure (13, 15, 26, 27). In general, the surgical decision in UPJOHN cases is made based on US findings. Therefore, accurate determination of hydronephrosis severity is very important for infants associated with UPJHN. In severe cases of hydronephrosis (SFU 4) with renal parenchymal thinning, clinicians should make a surgical decision without delay, as kidney function may also be impaired in a short time. Based on EAU and ESPU 2019 Guidelines on pediatric urology, surgical indications for UPJHN are impaired renal function (<40%), significant renal functional decrease (>10%) in control scans, poor drainage after furosemide injection, increased AP diameter, and SFU-III/IV (8, 26). Although there are problems with some of these indications, absolute surgical indications in the follow-up of UPJHN cases can be considered as renal parenchymal thinning (<3 mm), contralateral kidney balancing hypertrophy and decreased kidney function. Differential renal uptake on diuretic renography <30% in unilateral cases and <35% in bilateral cases is usually required a surgical intervention. Surgical treatment can also be recommended in children whose SFU3 hydronephrosis continues for 3 years and develops compensated hypertrophy in the contralateral kidney (27). If the main goal during conservative monitoring is to protect the child from the risk of permanent kidney damage, waiting for ultrasonographic or functional deterioration is a cornerstone that must be distinguished very carefully in each case. It should be noted that at this cornerstone, the presence of symptoms such as recurrent UTI, hematuria, kidney stones or pain will speed up the decision of surgical intervention (21, 26, 27).

As mentioned above, the clinical picture of UPJHN should be evaluated in two different categories, considering that most cases are asymptomatic and diagnosed on routine prenatal US screening; (a) asymptomatic infants who are usually managed conservatively (b) children who present at an older ages with urinary symptoms or as a result of incidental findings during the analysis of unrelated problems.

## Infants With Prenatal Diagnosis

Symptomatology in a newborn with antenatally diagnosed UPJHN is usually the absence of symptoms. However, the most frequent symptom of UPJHN in neonates and infants was a palpable flank mass in the past. Most of the abdominal masses encountered in the neonatal period are related to hydronephrotic kidneys. Therefore, a palpable abdominal mass may be the first finding to be considered in a physical examination in a newborn with UPJHN.

Since UPJHN is often associated with other congenital anomalies, including imperforated anus, contralateral multicystic kidney, congenital heart disease, VATER syndrome, and esophageal atresia, in a newborn with established prenatal diagnosis, a thorough examination of all systems should be performed (8). Occasionally, UPJHN can also be diagnosed during extended diagnostics of other congenital abnormalities. On the other hand, in all children with a diagnosis of urinary tract infection (UTI) within the early neonatal period, urinary tract obstruction, UPJHN should also be considered.

### Urinary Tract Infection

Children with UPJHN and impaired urinary drainage are considered to be prone to severe UTIs (28, 29). Although UTI is an uncommon presentation in UPJHN cases with an incidence of 1.3–12%, it may be quite severe requiring urgent intervention and drainage (4, 30–35). Previous reports suggest that the risk of UTI increases with the degree of hydronephrosis, and patients with high-grade hydronephrosis have significantly higher UTI rates than those with mild hydronephrosis (13.8 vs. 4.1%) (36–39). Although the studies are not standardized in terms of the use of prophylactic antibiotics, the method of detecting infection or the selection of patients for VCUG, it has been clearly demonstrated that patients with mild or moderate hydronephrosis are at much lower risk of significant UTI than patients with severe hydronephrosis.

When a child with UPJHN applies with a febrile UTI, the possibility of associated VUR is an important issue to consider. Based on the fact that some studies show one-third of cases having a VUR (8, 40, 41); VCUG is often favored by European guidelines for all children with UPJO (42, 43). Before deciding to apply VCUG, an invasive procedure with radiation exposure in UPJHN patients, it should be taken into account that in many cases that are often asymptomatic, VUR may improve over time and the concept of benefit-harm to the patient (44–46).

Madden et al. (47) performed VCUG in more than 80% of their patients with UPJHN and in no case detected VUR. In the same study, it was reported that patients who did not undergo VCUG remained asymptomatic and no imaging was required except for follow-up ultrasounds (47). Given the low rate of UTI reported, it may be considered that antibiotic prophylaxis has a limited role in the management of such patients (13, 47–49), and VCUG screening is considered to be optional (50). However, more aggressive evaluation and intervention, including antibiotic prophylaxis and VCUG are often indicated in those with worsening or high-grade hydronephrosis (47, 51–53). It should be noted that the presence of ureter dilatation is also important to suspect VUR even in severe hydronephrosis cases.

Another issue that can be considered for the prevention UTI in boys with UPJHN may be circumcision. Ellison et al. (54) reported that the risk of UTI in boys with UPJHN decreased significantly when circumcised. Although there may be no direct relationship since the stasis is in renal pelvis away from the external urethral meatus, in clinical practice, circumcision may be recommended for infant boys who have UTI history.

## Children Without Prenatal Diagnosis

Unlike asymptomatic presentation early in life, older children with UPJHN are often diagnosed due to their specific or non-specific symptoms. A carefully gathered clinical history played a very important role in the diagnosis of patients with UPJHN. These symptoms are usually febrile UTIs, a palpable mass, or unexplained abdominal or flank pain. In addition, UPJHN can be detected during evaluation of stone disease and sudden onset hypertension (8). Another small group ordered for a completely unrelated issue during imaging is diagnosed by chance.

### Pain

In children with UPJHN/UPJO, pain is primarily the result of dilation, stretching and spasm of the urinary tract, when the urine flow exceeds the capacity to drain properly. The causes of pain are generally muscle spasm, increased proximal peristalsis, local inflammation, irritation and edema at the site of obstruction. It develops through chemoreptor activation and stretching of the submucosal free nerve endings. The severity of pain depends on the individual's pain threshold and perception, and on the speed and degree of changes in hydrostatic pressure within the proximal ureter and renal pelvis. Chronic severe obstruction usually does not cause pain.

Although it is generally thought to have gastrointestinal symptoms, It should be noted that attacks of unexplained recurrent vomiting or abdominal discomfort may be associated with UPJ obstruction in infants (55). Sudden onset of severe abdominal pain, nausea, and vomiting, often in the late evening, is typical in older children with UPJO. This colicky-type pain usually begins in the upper lateral midback over the costovertebral angle and occasionally subcostally. It radiates inferiorly and anteriorly toward the groin. At their initial presentation, this symptomatology is far more common than febrile urinary tract infections or hematuria (8, 56). Pain along with increased diuresis should also raise the level of suspicion for an obstructive process. This usually occurs in children who receive a diuretic challenge during a furosemide renal scan.

It is important to recognize that patients with extrinsic anatomic abnormalities (e.g., lower pole crossing vessels) can present with colicky flank pain, which is sometimes associated with vomiting, and may present misleadingly unremarkable test results during their asymptomatic periods (56, 57). There is no history of hydronephrosis in the neonatal period In 75–100% of children with crossing vessels (57–59). The incidence of colickly pain in pure extrinsic UPJHN has been reported as 71.8–100%, increasing with age (57–59). The average age of patients with a crossing vessel is between 7 and 11 years and is statistically higher than in patients with pure intrinsic obstruction (58–61). An ultrasonography performed in the symptomatic period can prevent delay in diagnosis of extrinsic UPJHN due to crossing vessel.

### Urinary Stone Disease

**Figure 1:**
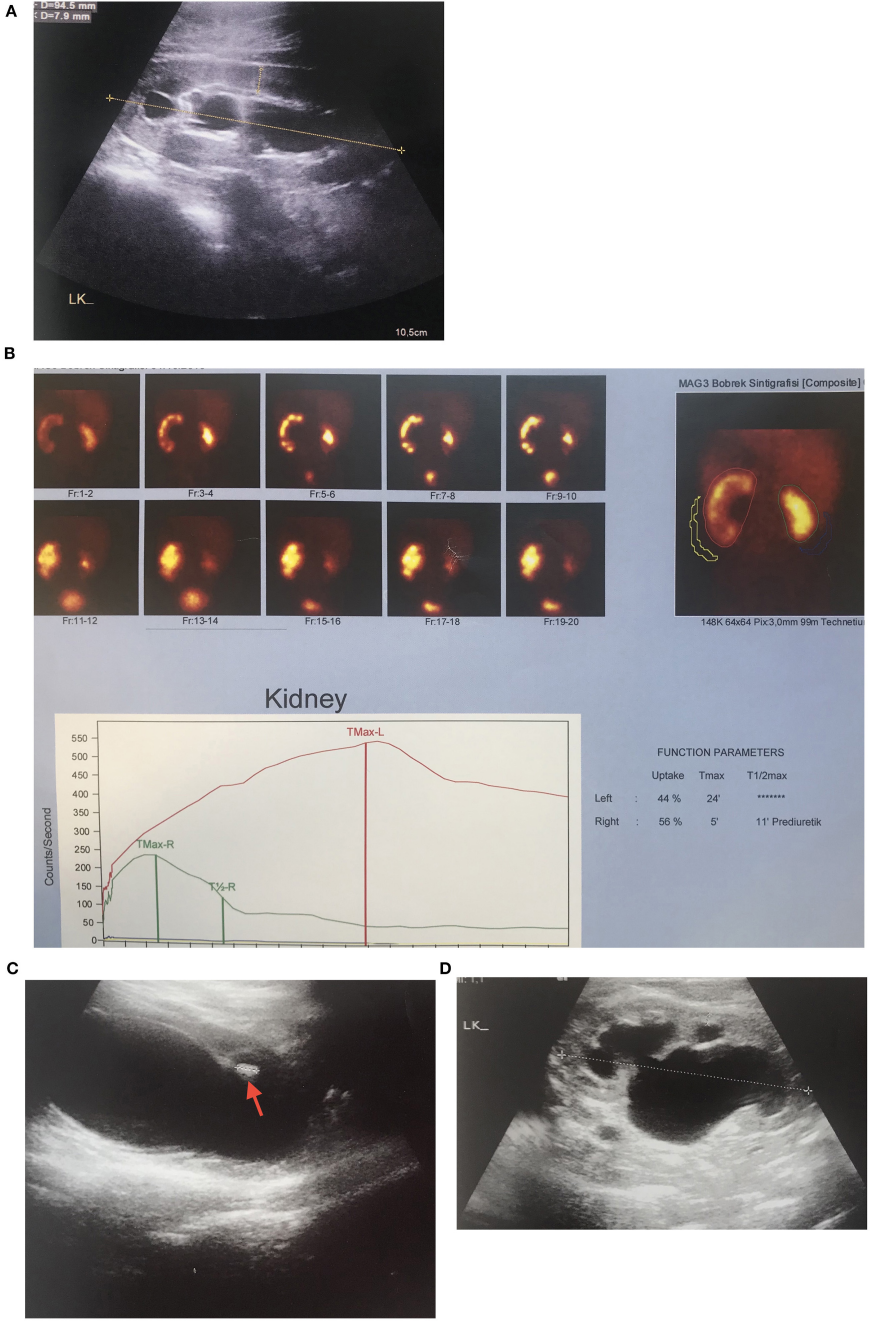
UPJHN in 3 months-old boy with prenatally detected left hydronephrosis **(A)** Severe hydronephrosis with 2.4 mm paranchymal thickness and 22 mm in AP diameter of pelvis renalis **(B)** Left obstructive hydronephrosis with 44% of differential function on MAG 3 scintigraphy **(C)** Mobile hyperechogenic particules in renal pelvis and calyces, hyperechogenicity in the lower calyces which are suggested urinary stone formation **(D)** Mild hydronephrosis with 8 mm paranchymal thickness and 16 mm in AP diameter of pelvis renalis in 9 years after left pyeloplasty.

Hydronephrosis is considered as a risk factor for stone formation in children. Although the etiology of stone formation does not depend solely on the pelvicaliceal anatomy, impaired urinary drainage, decreased or abnormal peristalsis, increased urine transit times and larger pelvicaliceal volumes play a subtle role during the beginning of the nucleation process in UPJHN patients with nephro/urolithiasis (62) (Figure 1).

### Hypertension

Published pediatric reports of hypertension obviously caused by hydronephrosis are few, and the numbers of patients included in these reports are very low (63–68). On clinical basis, the number of cases diagnosed with UPJHN/UPJO by referring to the results or symptoms of high blood pressure in the child age group is very few. While the development of clinically significant hypertension or proteinuria is very rare in patients with unilateral hydronephrosis, the same is not the case for bilateral disease (8, 63). Depending on the onset, level, and degree of obstruction as well as the presence of renal parenchymal damage or dysplasia, hypertension may develop during the follow-up.

**Figure 2:**
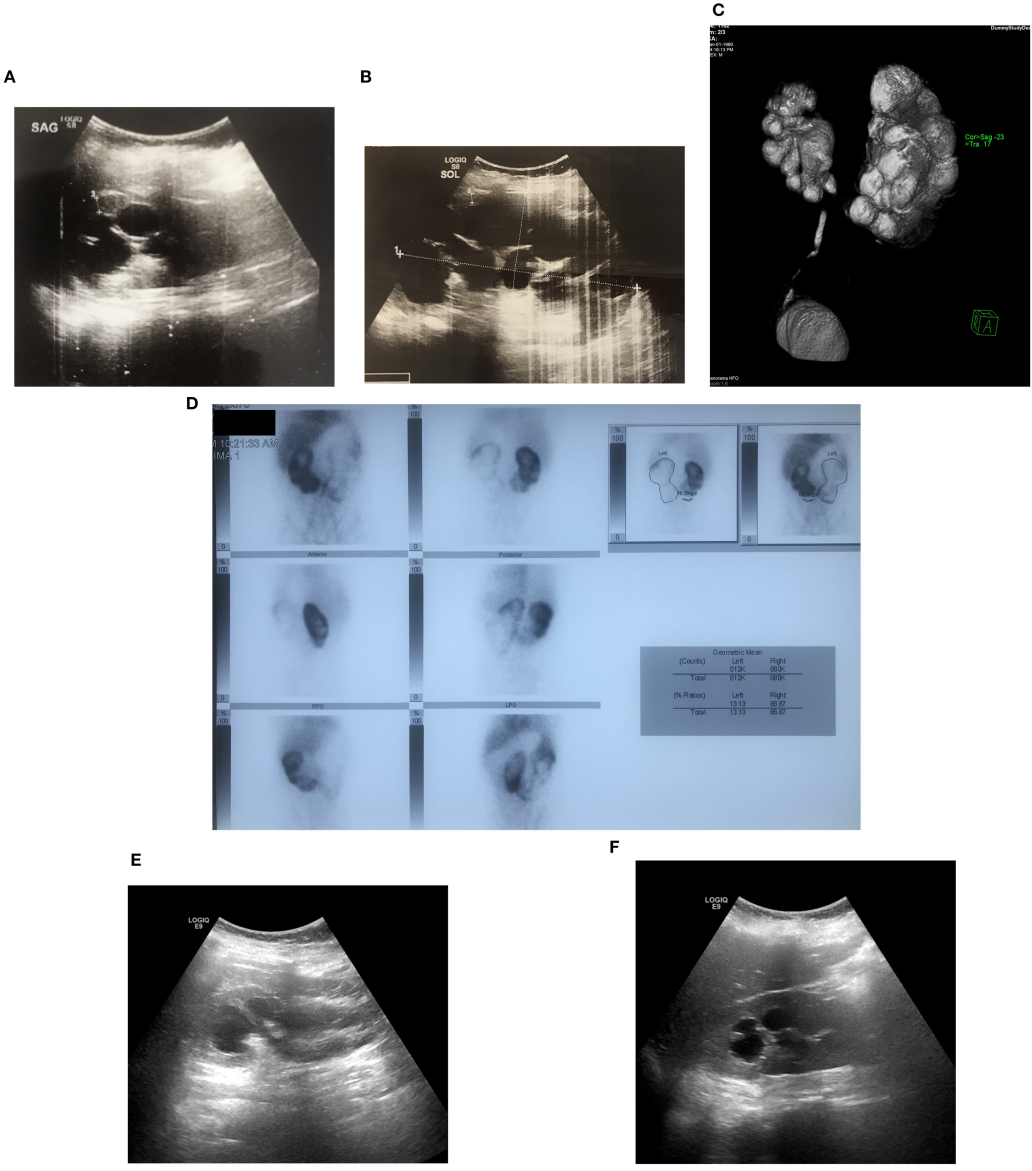
Bilateral UPJHN in 12 years-old boy with prenatally detected bilateral hydronephrosis with no follow-up who presented with severe hypertension and high serum creatinine (1.6 mg/dl) **(A,B)** pre-operative US images showing right and left severe hydronephrosis **(C)** Severe renal paranchymal loss on left kidney with 13% of differential function on DMSA scintigraphy **(D)** bilateral obstructive UPJ type hydronephrosis shown by MR urography **(E,F)** post-operative (bilaterally pyeloplasty) US image showing a resolution of left and right hydronephrosis, which was followed by resolution serum creatinine (0.67 mg/dl) and hypertension.

It has been demonstrated that the function of the hydronephrotic kidney is rather well-preserved in young children, therefore it appears that the intrarenal mechanism leading to hypertension is also reversible (6, 11). The clinical importance of such finding is that surgical management may prevent the development of chronic hypertension and associated comorbidities in patients with severe hydronephrosis (68–70). The pediatric urologist and nephrologist may have to pay more attention to the risk of development of high blood pressure in patients with severe hydronephrosis (Figure 2).

## Conclusion

Current management approach for most children with UPJHN is often considered conservative follow-up because hydronephrosis associated with UPJ anomalies can safely improve over the time. However, it is clear that delayed decision making in the case of obstructive hydronephrosis, which requires surgical intervention, leads to impaired kidney function and long-term morbidity.

It should always be kept in mind that clinical and symptomatological findings, as well as radiological tests, should be carefully evaluated so that the conservative follow-up strategy does not put a single patient at risk for possibly irreversible kidney damage.

Although conservative management algorithms and surgical indications are still an ongoing problem and there is no consensus among different disciplines, it is very important to maintain a long follow-up in both conservatively managed and surgical cases, taking into account negative prognostic factors.

## Author Contributions

The author confirms being the sole contributor of this work and has approved it for publication.

## Conflict of Interest

The author declares that the research was conducted in the absence of any commercial or financial relationships that could be construed as a potential conflict of interest.
